# Damage Propagation Analysis in the Single Lap Shear and Single Lap Shear-Riveted CFRP Joints by Acoustic Emission and Pattern Recognition Approach

**DOI:** 10.3390/ma13183963

**Published:** 2020-09-07

**Authors:** Claudia Barile, Caterina Casavola, Giovanni Pappalettera, Paramsamy Kannan Vimalathithan

**Affiliations:** Dipartimento di Meccanica Matematica e Management, Politecnico di Bari, Via Orabona 4, 70125 Bari, Italy; casavola@poliba.it (C.C.); giovanni.pappalettera@poliba.it (G.P.); pk.vimalathithan@poliba.it (P.K.V.)

**Keywords:** CFRP joints, hybrid joints, acoustic emission (AE), pattern recognition technique, k-means++

## Abstract

An innovative way of using the Acoustic Emission (AE) technique is introduced in this research work. The ratio of recorded acoustic energy and the counts recorded for each acoustic event were used for characterizing Carbon Fiber Reinforced Plastic (CFRP) laminates adhesively bonded with and without mechanical fasteners. The cumulative counts and cumulative energy of the recorded acoustic events were used for identifying the critical points of failure under loading of these hybrid joint specimens. The peak amplitude distribution was used for identifying the different damage modes such as delamination, matrix cracking and fiber breakage, albeit, ineffectively. The new parameter energy per count was introduced in this work, which can successfully identify the different damage modes under loading. To differentiate the damage modes using the energy per count, they were clustered using k-means++ pattern recognition technique. The method introduced in this work can estimate the damage modes of the CFRP specimens.

## 1. Introduction

The feasibility of using composite materials in larger structures is one of the major reasons for its tremendous growth over the last few decades. The need to join structural composites in a larger structure is inevitable. However, joints are considered potentially the weakest link of a structure. Thus, it becomes essential to study the strength and predict the failure of the joints [1,2]. Composite structures can be joined mechanically by using mechanical fasteners or bonded adhesively. The combination of these two methods, typically known as ‘hybrid’ can also be used for joining structural composites.

Adhesive bonded joints have an inherent advantage over the mechanical fasteners; they can distribute the load more efficiently and reduce the stress concentration [3,4]. The adhesive bonded joints can also reduce the cost and time of repairing the structures. However, for repairing the adhesively bonded joints, the process parameters must be controlled cautiously. Improper curing can cause detrimental effects on the joints, which may even lead to catastrophic failures.

To overcome this problem, the combination of adhesive bonded joints and mechanical fasteners can be used. Although few reports suggest that the hybrid joints cannot achieve significant improvement in structural properties over a well-processed, adhesive bonded joints, the mechanical fastening can be employed as a safeguard against the internal defects of the adhesive layer and prevent premature failure [5,6]. In other words, hybrid joints do not provide significant advantage over the adhesive joints in a structure but can prevent defect/damage propagation [7].

The premature failure or damage propagation in composite structures, especially the ones which releases less fracture energy such as delamination or microcracking in the matrices cannot be easily identified by using conventional Nondestructive Evaluation (NDE) techniques [8,9,10]. Several sensor types have been employed for the NDE evaluation of reinforced materials. The details about the capacitive and resistive sensors used for this purpose and their applications can be found in literatures [11,12]. The Acoustic Emission (AE) technique is rather efficient in identifying these damages, which can be attributed to its efficacy in sensing transient elastic waves [13]. The AE sensors can record the transient elastic waves, which are generated by sudden release of energy, when a material is strained and active defects are generated as a consequence. The AE is a passive NDE technique, which can provide information about the damage propagation in a material during its entire loading history.

The AE descriptors, total number of hits or events, counts, acoustic energy, peak frequency/amplitude are commonly used by researchers to identify/predict the damage propagation or initiation in a material. More details about the different AE descriptors and their applications can be found elsewhere [14,15]. The definitions of the AE descriptors considered for this study are provided in Table 1.

### Research Significance

Several researchers over the past decade have presented various methods of using AE descriptors for damage characterization in CFRP. It is difficult to discuss each of these research groups’ significant contribution and enumerate them. Nonetheless, several review works, which summarizes the importance and applications of different AE descriptors can be found in literatures. Particularly, Saeedifar and Zarouchas [16] have summarized the number of papers published each year, from 1975 onwards, on the damage assessment of laminated composites using the AE technique. In their research work, the damage initiation detection, damage identification and localization using AE technique by several research works has been summarized. Fotouhi et al. [17] used a criterion based on amplitude and energy of the AE events for damage identification in thin-ply laminated composites. In our review paper, the authors have summarized all the innovative methods of using different AE descriptors for the identification of damage in fiber reinforced polymer composites [18].

Each of these AE descriptors used in the literature have their own disadvantages and limitations. The aim of this research work is to introduce a new parameter, which can overcome the shortcomings of using AE energy and AE counts separately. Thus, by introducing a parameter that combines both the AE energy and counts for identifying the damage initiation in fiber reinforced polymer composites.

The main objective of this research work is to characterize the failure modes and predict the damage initiation in single-lap adhesively bonded and riveted Carbon Fiber Reinforced Plastics (CFRP) specimens.

In this research work, apart from using typical AE descriptors such as peak amplitude, cumulative AE energy and cumulative counts, the authors have introduced a new parameter known as energy per count. This parameter is classified using k-means++ pattern recognition algorithm into different classes to particularly identify the damage initiation site under loading. Detailed description about each of these parameters and the k-means++ pattern recognition algorithm is presented in the subsequent sections.

## 2. Materials and Methods

### 2.1. Materials

The CFRP prepreg laminate is prepared by impregnating high strength carbon fibers in ER450 epoxy matrix (SATTI CIT CC206 ER460, CIT Composite Materials, Legnano, Italy). The resin percentage in the composite laminate is 43% and each ply has a nominal ply thickness of 0.244 mm. The carbon fibers are stitched in a woven configuration with the layers of fibers overlapping one another. The laminates are cured by autoclave method. The adhesive used for bonding the laminates is an epoxy adhesive EA9395 with a shear strength of 25 MPa and a peel strength of 65 MPa. The adhesive is applied to the laminates and are cured at an elevated temperature of 100 ℃ to 150 ℃ for 1 h. Two types of configuration of specimens are prepared for this study.

The adhesively bonded Single Lap Shear (SLS) and hybrid Single Lap Shear with Rivets (SLS-R) are prepared using the aforementioned methods. The geometry of the specimens, the number of plies, orientations are given in Table 2 and Table 3.

**Figure 1:**
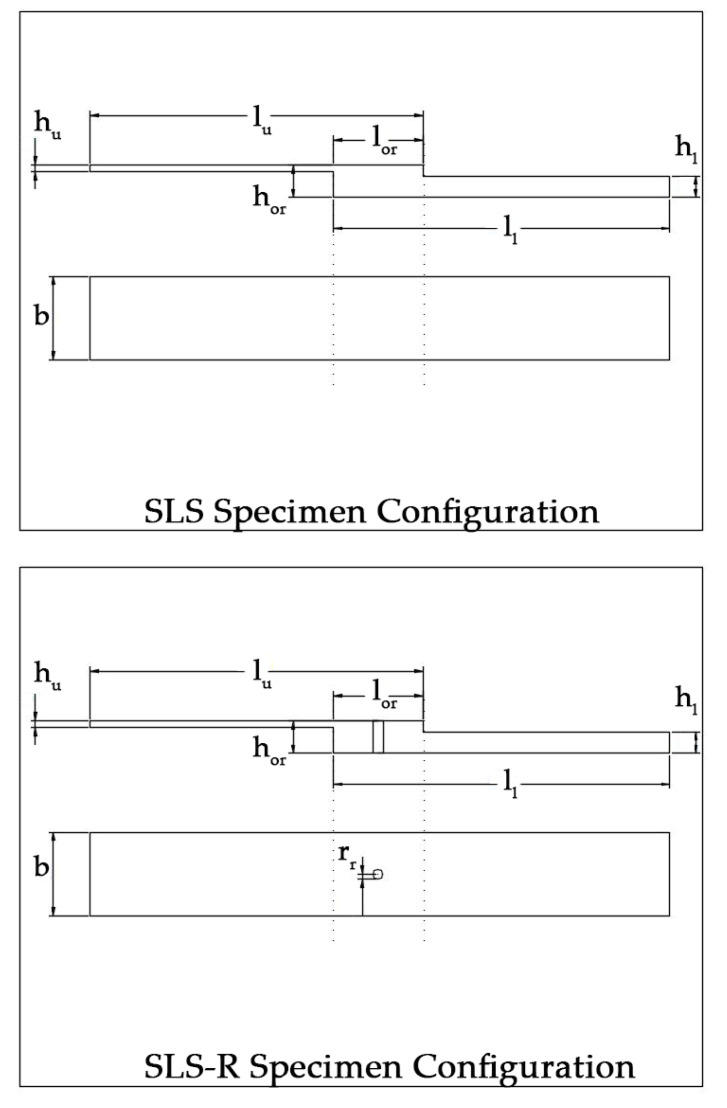
Configuration of SLS and SLS-R Specimens.

For SLS-R specimens, a hole was drilled in the middle of the adhesive overlapping region. The self-piercing rivets (SPR) are not used for this study because the adhesive thickness makes the specimen more brittle and SPR is not suited for those joints. A flat-headed aluminum rivet with a radius (*r_r_*) of 1.6 mm and a length of 14 mm is used for joining the SLS-R specimens. The configuration of the SLS and SLS-R specimens is presented in Figure 1.

### 2.2. Testing Methods

**Figure 2:**
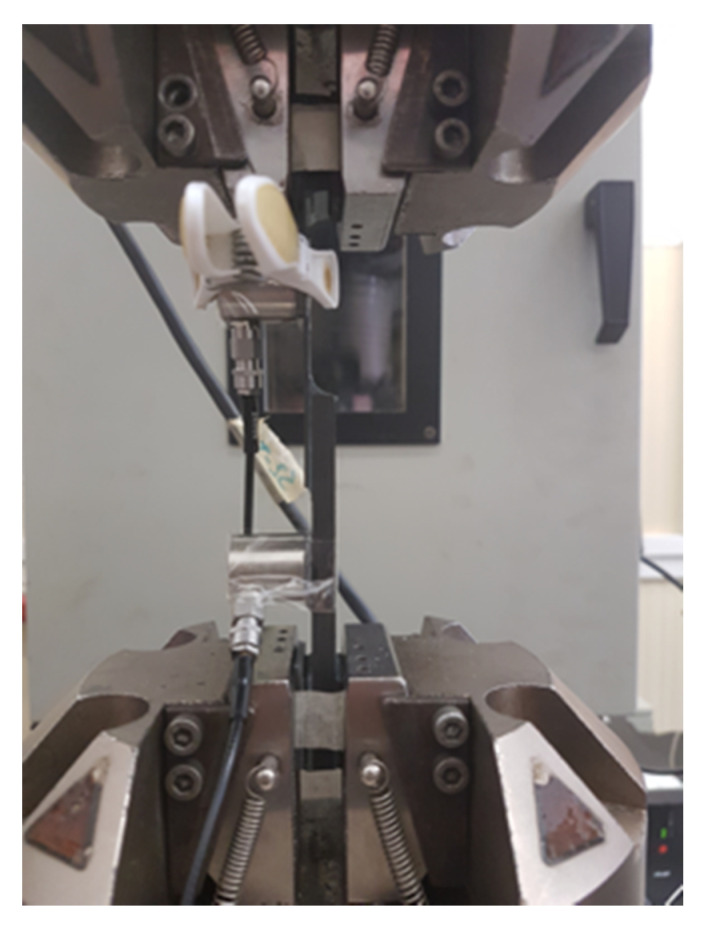
Testing setup with SLS specimen mounted along with the Acoustic Emission (AE) sensors.

There is no standard testing procedure for testing single lap joint specimens with larger thickness. Nonetheless, the standard testing procedure for ASTM D5868—Standard Test Method for Lap Shear Adhesion for Fiber Reinforced Plastic (FRP) Bonding is followed [19]. The tests are carried out in INSTRON Servo-Hydraulic testing machine with a load capacity of 100 kN under displacement controlled mode at a rate of 13 mm/min. The crosshead displacement speed of 13 mm/min is recommended by the aforementioned ASTM standard and also in the previous research works [12]. The tensile load is applied to the specimen. It has been indicated in several reports that the thickness of the adherend does not significantly affect the load distribution under displacement-controlled mode. The test setup with the specimen and the sensors mounted is presented in Figure 2.

### 2.3. Acoustic Emission Setup

For recording the acoustic signals emitted during loading, two narrowband sensors R30α (Physical Acoustics, MISTRAS Group, NJ, USA) are used. The sensors have an operating frequency range of 150 kHz to 400 kHz, with a resonant frequency of 300 kHz. It has been indicated in several reports that the AE signals will not be above 400 kHz in Fiber Reinforced Plastics (FRP) [20,21]. The narrowband sensor is selected based on that observation and to reduce the recording of noise signals. The sensor signals are amplified by 40 dB using a 2/4/6 AE preamplifier. The threshold for acquisition is set as 35 dB and the signals are recorded at a sampling rate of 1 mega samples per second (1 MSps) [22].

The sensors are mounted on the specimen at 40 mm from its center. Silica gel is interposed between the sensor surface and the specimen surface to enhance the coupling between the two elements. The silica gel also reduces the recording of reverberated signals arising from the surface of the specimen.

**Figure 3:**
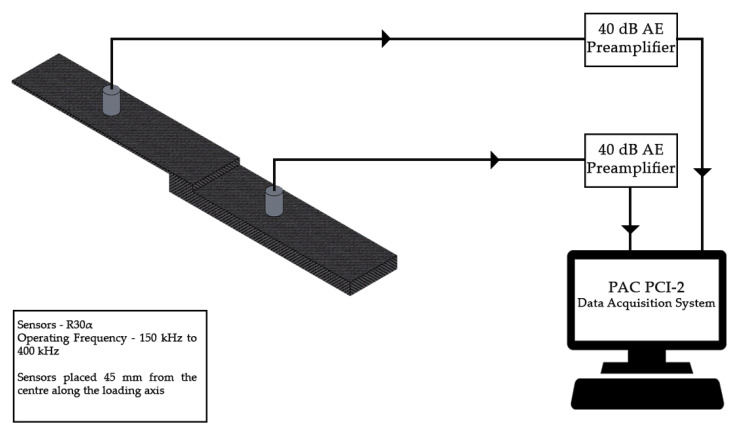
Schematic of Acoustic Emission Setup.

The distance of the sensor from the acoustic source can affect the recorded AE signals. For this reason, the attenuation of AE signals along the length of the specimen is studied using pencil lead break test and the attenuated values are fed to the PAC PCI-2 data acquisition system. The schematic of the AE setup is presented in Figure 3.

### 2.4. Acoustic Emission Descriptors

This section details the acoustic emission descriptors used for this study. The acoustic emission descriptors used for this study are peak amplitude, cumulative number of counts and cumulative energy.

Peak amplitude represents the largest voltage peak (U_max_) in the recorded AE signal waveform with respect to the reference voltage (U_ref_) [18,23]. The reference voltage is adjusted by the data acquisition system depending on the amplitude threshold provided. The peak amplitude (*A*) can be written in an equation form as,

(1) $$A=20\log\left(\frac{U_{\max}}{U_{\mathrm{ref}}}\right).$$

The acoustic count (C) is the number of instances the amplitude of the signal crosses the threshold voltage in the recorded acoustic waveform. The acoustic energy is calculated as the integration of the square of the recorded transient voltage (U_i_) over the time period of the signal t_0_ to t_1_ [24]. The acoustic energy (E_AE_) can be written in equation form as,

(2) $$E_{\mathrm{AE}}=\;\int_{t_{0}}^{t_{i}}U_{i}\left(t\right)\mathrm{dt}.$$

In addition to these parameters, energy per count is also taken for this study. It is the ratio of total energy of the acoustic signal recorded to the number of counts in each acoustic event. Normally, the AE signals are classified purely based on the energy, however, under certain circumstances, the results can be misleading [25]. For instance, fiber breakage in a FRP release AE waves with large E_AE_, at the same time, fiber matrix debonding or matrix cracking can produce large number of counts. This is because the acoustic waves propagate in two different modes—lower order symmetric and higher order asymmetric. The higher order symmetric waves, originating from failures such as fiber breakage carries large energy and short duration, consequently, a lower number of counts. The lower order asymmetric waves originating from matrix cracking or debonding carries large number of counts with lower energy. In some cases, when the material has high stiffness/brittleness, the cumulative number of energy recorded from these two types of modes can be similar. This makes them hard to categorize and makes it difficult to identify the damage modes. In this work, this problem has been addressed through a very simple yet innovative approach—energy per count. Using this parameter, it is easy to categorize the different signals with their energy levels, which will be helpful in identifying the damage mode in the material. In simple terms, the energy per count (E_C_) can be expressed as, for each acoustic event ‘N_i_’,

(3) $$E_{C}=\frac{E_{\mathrm{AE},i}}{C_{i}},$$

where i represents number of the acoustic event ‘N’.

### 2.5. Data Clustering Using k-means++ Pattern Recognition

The recorded AE descriptors are used for characterizing the damage progression in the tested specimens. The ratio of acoustic energy and count is taken as energy per count. The calculated energy per count is clustered using k-means++ algorithm. For considering the optimum number of clusters in which the data can be classified, Davies-Bouldin Index (DBI) is used [26,27]. The DBI index for cluster ‘k’ ranging from 1 to 6 is calculated in MATLAB^®^. The cluster with the minimum number of DBI is considered as optimal.

The algorithm for the k-means++ clustering is briefly explained in this section. More detailed information about using this algorithm and the advantages and limitations of this algorithm over other data clustering algorithms can be found elsewhere [28,29].

The dataset to be clustered into predefined number of classes ‘k’ is taken as X.

*Step. 1*  Select a random point from the input dataset X. This random datapoint is considered as the first centroid (c_1_).
*Step. 2*  Compute the distance of all the datapoints from the centroid c_1_. The distance between the centroid c_j_ and each datapoint m is stored as d(x_m_,c_j_).
*Step. 3*  The next centroid is selected with the following probability in random from the dataset X.

(4) $$\frac{d^{2}\left(x_{m},c_{1}\right)}{\sum_{j=1}^{m}d^{2}\left(x_{j},c_{1}\right)}$$

*Step. 4*  Choose center j by computing the distance between each datapoint of each dataset and the respective centroid.
*Step. 5*  Assign each datapoint to the closest centroid.
*Step. 6*  Repeat Steps 4 and 5 until all centroids k are chosen.
*Step. 7*  Calculate datapoint to cluster centroid distance for all the datapoints with respect to their assigned centroid.
*Step. 8*  Calculate the average of the datapoints in each cluster to obtain new (or optimal) centroid locations.
*Step. 9*  Repeat Steps 7 and 8 until the cluster assignments do not change (or the maximum number of iterations is reached).

A centroid is assigned for each predefined number of clusters and all the datapoints from the dataset X is assigned to each of the cluster based on the shortest distance between the datapoint and the centroid.

## 3. Results and Discussions

### 3.1. Mechanical Test Results

**Figure 4:**
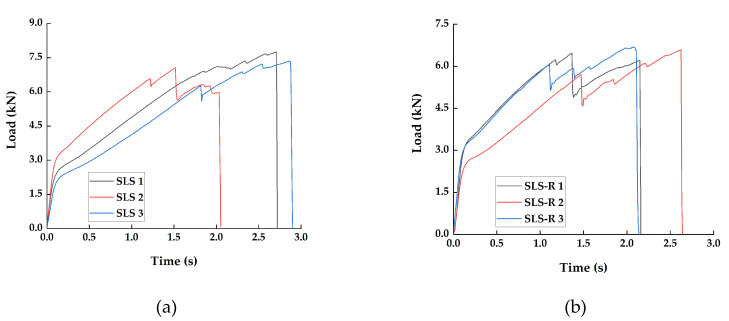
Load response plotted over time for the (**a**) SLS Specimens SLS 1, SLS 2 and SLS 3 and (**b**) SLS-R Specimens SLS-R 1, SLS-R 2 and SLS-R 3.

For this study, three specimens each from SLS and SLS-R group are tested. The tensile load is applied on the specimens at a crosshead displacement of 13 mm/min and the load-time response is presented in Figure 4. The load response is plotted against time instead of crosshead displacement is for the ease of comparing the mechanical results with the acoustic results, which are plotted over time.

Both the group of specimens have multiple load peaks before failure. It can be observed that the SLS specimens have carried more load than the SLS-R specimen group. It has been indicated by Chowdhury et al. that the riveted specimens do not necessarily have significant strength as the normally adhesively bonded specimen but can reduce the propagation of internal failure within the adhesive [5,6]. Typically, in an adhesively bonded joints, failure occurs in two modes. The first mode is interfacial failure or adhesive failure which occurs between the adhesive and the adherend. The second mode is the cohesive failure within the adhesive layer, which is due to the delamination of the composite adherend [8,9]. The two most significant stresses that induce the failure modes are—interlaminar stresses at the vicinity of the bond line edges which causes the interfacial failure and the normal stress acts on the adhesive, the peel stress, which is responsible for the cohesive failure. Generally, the peel stresses are higher compared to the interlaminar shear stresses and are responsible for the failure. Since the specimen groups SLS and SLS-R has significantly larger thickness (8.25 mm approx.), most of the failure could be attributed to the interlaminar failure [10].

The resulting peel stress probably has initiated the failure at the vicinity of the boundaries between the adhesive layer and the adherend at the edges. However, due to the high peel strength of the adhesive, the cohesive crack path is not very stable. This can be attributed to the multiple load peaks in both the specimen groups.

Moreover, the peel stress is typically larger at the center of the adhesive overlapping region and is lower near the boundary edges [30,31]. The SLS-R specimens are riveted at the center, where the peel stress will be maximum. This probably had made the crack growth more unstable, resulting in multiple load peaks in the SLS-R specimens. From Figure 3, it can be observed that the load peaks in SLS-R specimens occur earlier and are also lower than in SLS specimens. The average peak load of SLS group of specimen is 6.91 kN, whereas SLS-R specimens have an average peak load of 6.08 kN.

Generally, the yielding of the rivets is observed around 4 kN to 6 kN in many of the hybrid composites. Nonetheless, in all the previous studies, one of the adherend is a metal, which makes them a hybrid composite [32]. In this present study, both the adherends are CFRP, which are stiffer than their metal counterparts. This results in forming high stress concentration around the rivets which are propagated towards the boundary edges, leading to the failure. This could be another reason why the SLS-R group of specimens have lower peak load than SLS specimen group.

### 3.2. Acoustic Emission Results

#### 3.2.1. Cumulative Counts

**Figure 5:**
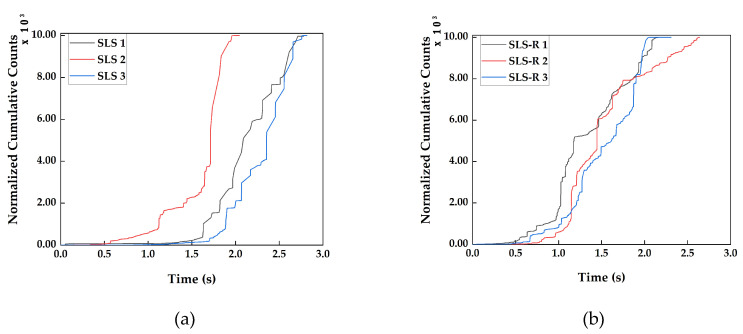
Normalized Cumulative Counts recorded for (**a**) SLS group of specimens, (**b**) SLS-R group of specimens.

Acoustic Emission Counts are commonly used for predicting the critical failure occurrences under loading. Counts have been used for more than four decades for predicting the failure in composite structure and pressure vessels [33]. However, under static loading, the absolute number counts recorded depends not only the failure phenomenon but also on how the sensors are placed on the specimen. So, it is difficult to compare counts as the only parameter for predicting the failure in an element. For this reason, the counts recorded for all the specimens, both SLS and SLS-R, they are normalized for comparison. The cumulative counts recorded are presented in Figure 5.

In the specimen group SLS, for SLS 1, the cumulative count remains below 100 until it reaches the duration 0.5 s. For specimens SLS 2 and SLS 3, the cumulative count remains to me very low until the duration 1.5 s and 1.75 s, respectively. After that, it increases linearly. The point at which the cumulative count increases rapidly for SLS 1, SLS 2 and SLS 3 are 1.1 s, 1.65 s and 1.75 s, respectively. If the cumulative counts curve in Figure 5a is compared with the load responses in Figure 4a, the first load drop can be observed after this rapid increase in the cumulative counts. The rapid increase in the cumulative count is an indication that a major damage is about to occur in the specimen.

The acoustic waves generated during the straining and failure modes propagate in two different modes as mentioned in Section 2.4. The lower order symmetric AE signals, which represent the matrix cracking or interfacial failure normally has large number of counts [14,25]. The accumulation of these damages before the specimens loses their load carrying capacity is the reason for the rapid increase in the cumulative counts before the failure. Thus, each time before a major failure is about to occur, the cumulative number of counts takes a steep increase indicating the occurrence of failure.

In Figure 5b, the similar pattern can be observed. The first rapid increase in cumulative counts can be observed at 1.0 s, 1.275 s and 1.2 s for specimens SLS-R 1, SLS-R 2 and SLS-R 3, respectively. While comparing with the load responses in Figure 4b, a pattern similar to the SLS group of specimens can be observed.

The major difference between the two group of specimens is the duration of the linear increase prior to the first rapid increase in the cumulative counts. The specimen group SLS has a longer duration of linear increase in the cumulative counts with respect to the SLS-R group of specimens. Moreover, before the rapid increase in SLS-R specimens, they also exhibit some small increases in the cumulative counts before the aforementioned duration of major failure occurrences. The differences in failure modes between the specimen groups SLS and SLS-R can be attributed for this phenomenon.

Both the specimen groups have larger thickness in the adhesive overlapping zone, close to 8.5 mm. As mentioned in the previous sections, the peel stress induced is significantly lower in both the specimen groups, which inhibit the cohesive failure of the composite. There will be more shearing of the adhesive layer before the cohesive failure starts to occur. In SLS-R group specimens; however, the presence of the rivets increases the stiffness of the specimen and induce more stress around them. This is the reason why there is less shearing, which is the reason for the shorter duration of linear increase in the cumulative counts in SLS-R group of specimens. This obvious stiffening effect is also evident from Figure 5a,b, as there are more steps in the increase in cumulative counts in SLS-R group of specimens than SLS group. As mentioned in Section 2.4, the cumulative increase in counts cannot be used solitarily for identifying failure modes in stiff specimens. For this reason, the cumulative increase in energy and peak amplitude are introduced in the subsequent sections.

#### 3.2.2. Cumulative Energy

**Figure 6:**
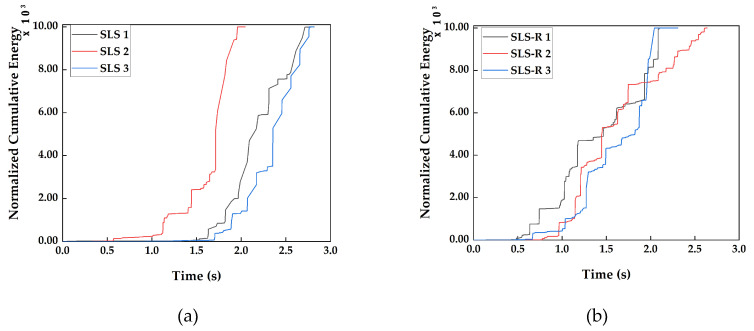
Normalized Cumulative Energy recorded for (**a**) SLS group of specimens, (**b**) SLS-R group of specimens.

It has been indicated by several researchers that there exists a linear relationship between the cumulative counts and cumulative energy [34,35,36]. This is evident while comparing Figure 5 with the cumulative energy recorded during the acoustic events, which is presented in Figure 6.

Both the cumulative counts and cumulative energy looks almost identical in comparison. However, while looking closely, some small differences can be observed. Particularly in the Figure 6b, the cumulative energy for the specimen groups SLS-R.

There is no linear increase in the cumulative energy for specimen SLS-R 1 and SLS-R 3; the energy remains almost close to zero until the durations of 0.5 s and 0.65 s, respectively, followed by a sudden increase. Moreover, the increase in normalized cumulative energy is larger than the increase in normalized cumulative counts at the same duration. This indicates that there is more energy released during these acoustic events than the number of counts. This means that the acoustic wave propagated in higher order symmetric modes, indicating the interfacial failure or fiber breakage. This could be an indication that the induced stress by the rivets may have induced either interfacial failure or fiber breakage at that time.

Regardless of this one particular section, most of the pattern in Figure 5 and Figure 6 are almost identical. This is the reason why the cumulative counts and cumulative energy are efficient in predicting the failure occurrence but cannot indicate the failure modes clearly. This compels the researchers to use different AE descriptors. In this research work, the peak amplitude and energy per count are used as well.

#### 3.2.3. Peak Amplitude Distribution

The peak amplitude has been used by many researchers for categorizing the failure modes in fiber reinforced composites [21]. It is one of the most debated parameters for characterizing failure modes. Many researchers come to a consensus that the peak amplitude is efficient in categorizing failure modes, only if it is used with one or more AE parameters.

**Figure 7:**
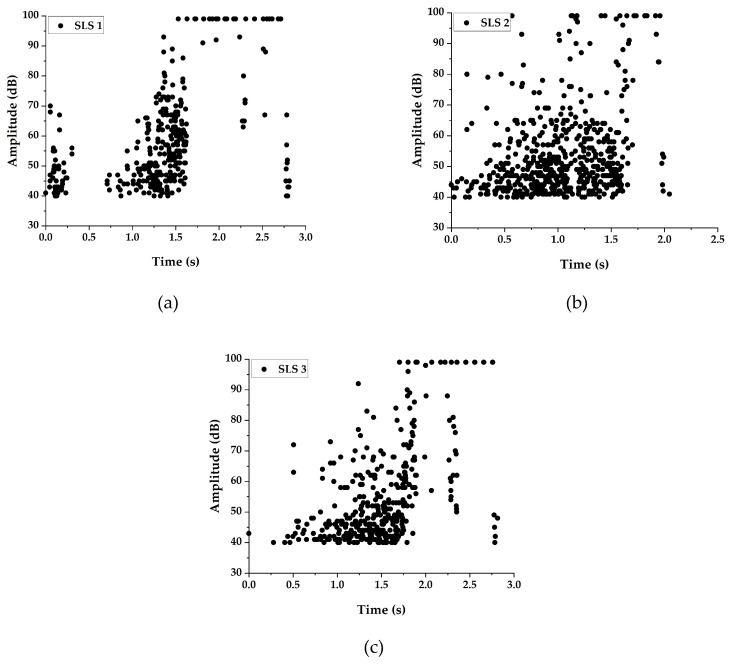
Peak Amplitude distribution for Specimens (**a**) SLS 1 (**b**) SLS 2 and (**c**) SLS 3.

**Figure 8:**
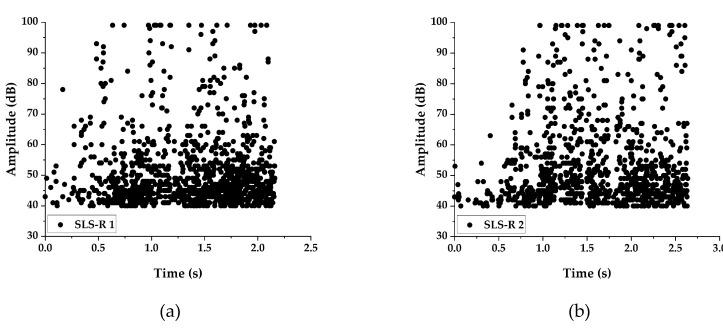

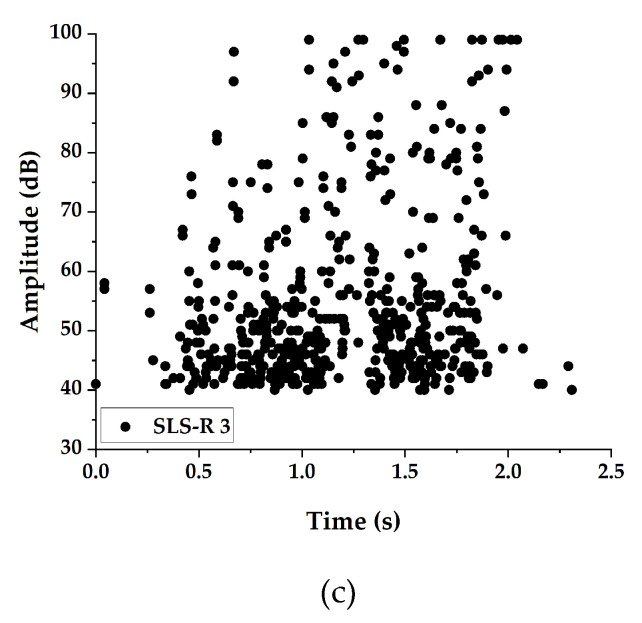
Peak Amplitude distribution for Specimens (**a**) SLS-R 1 (**b**) SLS-R 2 and (**c**) SLS-R 3.

The peak amplitude distribution recorded during the test of SLS and SLS-R groups of specimens are presented in Figure 7 and Figure 8, respectively.

The peak amplitude distribution between the specimens SLS 1, SLS 2 and SLS 3 are not similar during the initial stages of loading. At the early stages of loading, the specimen SLS 1 released acoustic waves with a peak amplitude distributed between 40 dB and 70 dB, which is followed by a period of acoustic gap. This is followed by a surge of acoustic events distributed between 40 dB to 75 dB. Once the specimen reached the duration of the initial peak load, as indicated in Figure 4a, acoustic events with an amplitude above 90 dB started to be observed. During the initial stages of loading, the SLS 1 specimen suffered severe matrix cracking and interfacial failure, which always releases AE energy below 65 dB to 70 dB. The acoustic gap indicates that the accumulated damage is about to cause a catastrophic failure. This can be observed by the sudden increase in AE events with amplitudes above 65 dB after the acoustic gap. This is also an indication why the SLS 1 specimen has suffered its initial failure at a very early stage (Figure 4a). The number of AE events prior to the initial damage has resulted in the consequential failure of the specimen.

However, it is not the same for specimen SLS 2, which has only a limited number of AE events at the initial stages of loading. Moreover, those AE events are well below 50 dB; indicating that there are very few matrix cracking or interfacial failure in the specimen SLS 2. Nonetheless, the AE events are distributed between 40 dB and 65 dB throughout the entire loading history until the specimen started to lose its load bearing capability completely. It must be understood that the matrix failure that occurred in the adhesive overlapping region, which is 8.5 mm thick may have released the AE waves with the distributed peak amplitude. But the specimen still transferred the load until the duration 1.2 s, where the major failure started to occur. It is from that instant, a higher number of AE events with amplitudes above 85 dB (or even 90 dB) can be observed.

Specimen SLS 3 has a unique trend as there are no AE events observed until 0.25 s duration. These differences in the failure modes can be attributed to the curing conditions of the adhesive applied. Moreover, the AE events above 85 dB normally represent fiber failure. However, in the present study, only a very few fiber failures are observed. Several researchers have indicated that the larger amplitude AE events can also represent the interlayer crack growth [25]. Since these specimens have suffered significant damage due to the cohesive crack growth, the high amplitude events in all the specimens can be directly attributed to the cohesive failure of the specimens.

The peak amplitude distribution in SLS-R group of specimens are identical to one another; however, they are entirely different from the amplitude distribution of SLS group of specimens. The important reason for this is because of the failed matrix rubbing with the pivoted rivets, creating more frictional waves. This the reason why the AE amplitude is distributed between 40 dB to 75 dB throughout the loading duration. The final stages of loading, however, are similar to the SLS group of specimens where the failure occurred due to the cohesive crack growth. The induced peel stress probably has caused the cohesive crack growth to propagate from the middle of the specimen to the upper adherend boundary, inducing the final failure of the specimen.

One important observation that can be observed while comparing the peak amplitude distribution, the SLS group of specimens show different trends, however, the SLS-R group shows similar trend. This means that the crack growth is unstable due to the larger thickness in the SLS specimens but is controlled and propagated in a controlled manner by adding the rivets. This coincides with the observations of Chowdhury et al. [5,6,37,38,39]. Nonetheless, even at the final stages of loading, the AE events are distributed in a wide range of peak amplitude, which makes it quite difficult to easily identify the failure mode. For this reason, a new parameter named ‘energy per count’ is used in this study.

#### 3.2.4. Energy per Count

**Figure 9:**
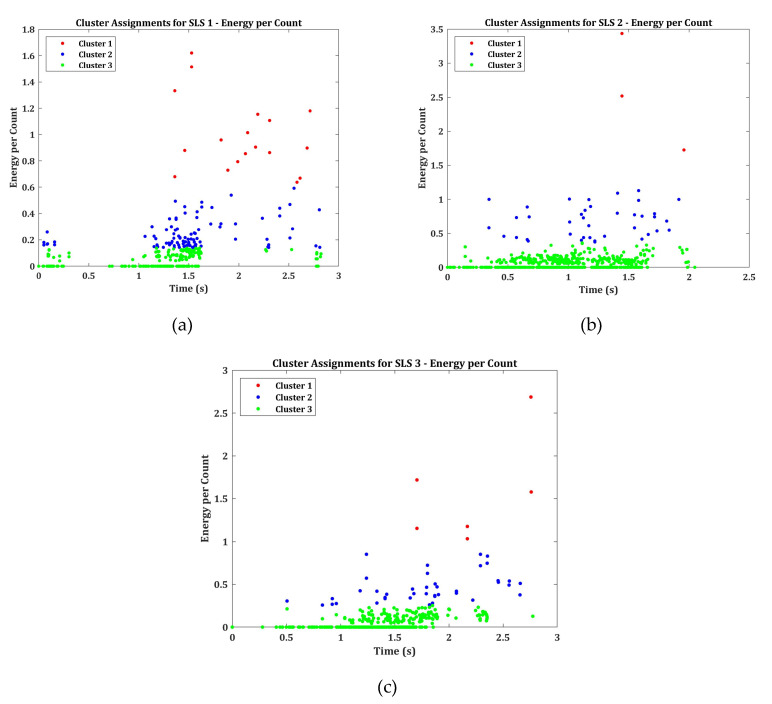
Clustered Energy per count for Specimens (**a**) SLS 1 (**b**) SLS 2 and (**c**) SLS 3.

The energy per count recorded for the SLS specimens are calculated using Equation (3) and are presented in Figure 9. The energy per count for all the specimens are clustered into 3 classes using k-means++ algorithm.

The clustered energy per count gives a clear indication of the failure modes in the specimen group SLS. The cluster 1 represent the AE signals with larger energy with lower number of counts, indicating the higher order symmetric AE signals. This means that the cluster 1 has AE signals representing the cohesive crack growth. The second cluster represents the AE signals released during the interfacial failure between the adhesive and the adherend. The cluster 3 possibly can represent the lower order asymmetric AE signals, which represent the matrix cracking.

In specimen SLS 1, a significant number of AE signals in cluster 1 can be observed even during the early stages of loading. This again coincides with the mechanical results from Figure 3 indicating why the specimen SLS 1 failed early and carried less load. The specimen SLS 2 and SLS 3, however, show a large number of events in cluster 2 and cluster 1. This means that the cohesive crack growth occurred in the SLS 2 and SLS 3 specimens probably have propagated along the thickness of the adhesive, resulting in the interfacial failure. This is the reason why many AE events above 90 dB can be identified in the later stages of loading in specimen group SLS 2 and SLS 3 in Figure 7b,c.

**Figure 10:**
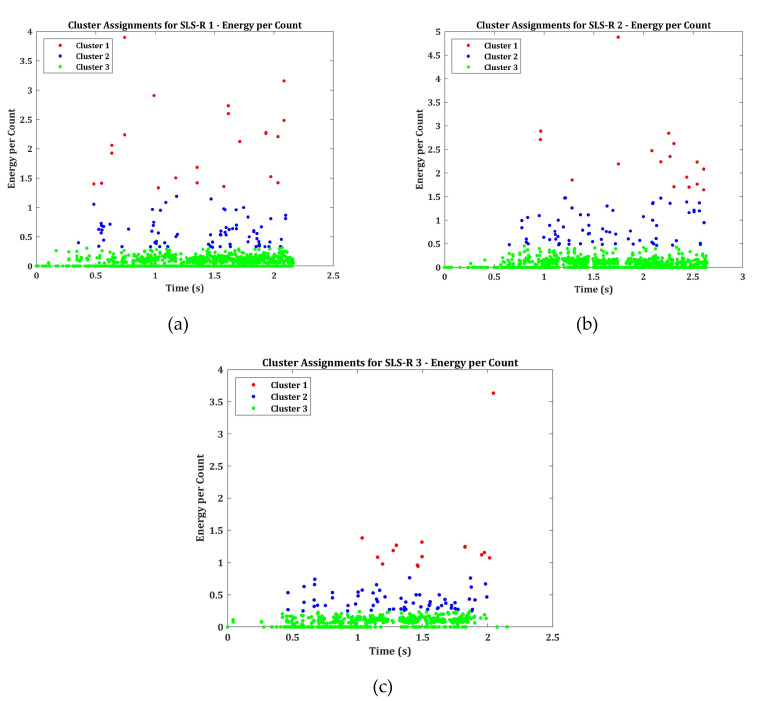
Clustered Energy per count for Specimens (**a**) SLS-R 1 (**b**) SLS-R 2 and (**c**) SLS-R 3.

The energy per count results of SLS-R groups of specimens are presented in Figure 10. As indicated in the previous section, the distributed AE amplitudes in SLS-R group of specimens might represent the friction between the damaged matrix and the rivets. This is evidently proved while looking at Figure 10. For all the three specimen groups, most of the AE events have the energy per count distributed in the cluster 3. Only a selective number of events can be observed in cluster 1 and cluster 2. This indicates that the events in cluster 3 represents—(1) matrix cracking and (2) friction between the rivets and the damaged matrix.

However, a lot of events in cluster 1 with a larger energy per count can be observed in SLS-R specimen group. This is because of the presence of rivets, which induce stresses around them which results in creating larger stiffness. When the material fails in the region of high stress, it releases AE events with larger energy per count.

One could easily be misled to the assumption that the events in cluster 1, 2 and 3 represents cohesive crack growth, through thickness crack growth and matrix cracking, respectively. This may be true only if more than one AE descriptor is used for analysis. This hypothesis is established by observing the results of normalized cumulative counts, energy and peak amplitude distribution and finally proved by the energy per count. Thus, it is always essential to use more than two AE descriptors to identify the damage mode.

## 4. Conclusions

The single lap adhesively bonded CFRP specimens with and without rivets are tested for this study. The damage modes are characterized using the Acoustic Emission technique. Different AE descriptors, namely cumulative counts, cumulative energy and peak amplitude are used for characterizing the failure modes in the specimens. The cumulative energy and cumulative counts can predict the major damage occurrences in the specimens, whereas the peak amplitude can categorize the failure modes. A new parameter named energy per count is introduced in this study. The energy per count recorded for acoustic events are classified into different clusters using k-means++ pattern recognition algorithm. The results from the clustered energy per count data efficiently identify the different damage modes in the specimens. Despite being an effective damage characterization tool, the AE technique, particularly the new parameter introduced in this study, will be more efficient if it can be compared with the fractographic studies or other stress distribution analysis using Digital Image Correlation or similar techniques. Nonetheless, the damage initiation sites can be monitored under the loading conditions effectively using the proposed method.

## Figures and Tables

**Table 1:** Definitions of basic Acoustic Emission (AE) descriptors used for this research work.

AE Descriptors	Definition
Acoustic Event	A local material change or straining that gives rise to an acoustic emission
Amplitude	The largest voltage peak in the recorded AE waveform, which is customarily expressed in decibels relative to the 1 µV preamplifier input
Counts	The number of times AE signal crosses the detection threshold
Energy	The total elastic energy released by the acoustic event which is above the detection threshold

**Table 2:** Single Lap Shear (SLS) Specimen Nomenclature, Geometry and Configurations.

**Upper Adherend**				
**Length *l_u_* (mm)**	**Width *b_u_* (mm)**	**Thickness *h_u_* (mm)**	**No. of Plies**	**Stacking Sequence**
101.6 ± 0.11	25.33 ± 0.12	1.3 ± 0.05	5	+45/+45/+45/−45/+45
**Lower Adherend**				
**Length *l_l_* (mm)**	**Width *b_l_* (mm)**	**Thickness *h_l_* (mm)**	**No. of Plies**	**Stacking Sequence**
101.6 ± 0.09	25.33 ± 0.14	6.4 ± 0.12	26	+45/[+45/−45]_12_/+45
**Overlapping Region**				
**Length *l_or_* (mm)**		**Width *b_or_* (mm)**		**Thickness *h_or_* (mm)**
26 ± 0.12		25.33 ± 0.25		8.5 ± 0.11

**Table 3:** Single Lap Shear with Rivets (SLS-R) Specimen Nomenclature, Geometry and Configurations.

**Upper Adherend**				
**Length *l_u_* (mm)**	**Width *b_u_* (mm)**	**Thickness *h_u_* (mm)**	**No. of Plies**	**Stacking Sequence**
101.6 ± 0.09	25.34 ± 0.10	1.3 ± 0.07	5	+45/+45/+45/−45/+45
**Lower Adherend**				
**Length *l_l_* (mm)**	**Width *b_l_* (mm)**	**Thickness *h_l_* (mm)**	**No. of Plies**	**Stacking Sequence**
101.6 ± 0.14	25.32 ± 0.16	6.4 ± 0.10	26	+45/[+45/−45]_12_/+45
**Overlapping Region**				
**Length *l_or_* (mm)**		**Width *b_or_* (mm)**		**Thickness *h_or_* (mm)**
25.40 ± 0.06		23.32 ± 0.12		8.27 ± 0.08
